# Development of pulmonary sarcoidosis in Crohn’s disease patient under infliximab biosimilar treatment after long-term original infliximab treatment: a case report and literature review

**DOI:** 10.1186/s12876-021-01948-6

**Published:** 2021-10-12

**Authors:** Shin Kashima, Kentaro Moriichi, Katsuyoshi Ando, Nobuhiro Ueno, Hiroki Tanabe, Sayaka Yuzawa, Mikihiro Fujiya

**Affiliations:** 1 Division of Metabolism and Biosystemic Science, Gastroenterology, and Hematology/Oncology, Department of Medicine, Asahikawa Medical University, 2-1 Midorigaoka-higashi, Asahikawa, Hokkaido 078-8510 Japan; 2 Department of Diagnostic Pathology, Asahikawa Medical University Hospital, 2-1 Midorigaoka-higashi, Asahikawa, Hokkaido 078-8510 Japan

**Keywords:** Crohn’s disease, Sarcoidosis, TNF-inhibitors, Original infliximab, Infliximab biosimilar

## Abstract

**Background:**

Inflammatory bowel disease (IBD) is chronic inflammation of the gastrointestinal tract, although its etiology has largely been unclear. Tumor necrosis factor inhibitors (TNF-I) are effective for the treatment. Recently, biosimilars of TNF-I, such as CT-P13, have been developed and are thought to possess equal efficacy and safety to the original TNF-I. Sarcoidosis is also a systemic granulomatous disease of unknown etiology. In steroid-resistant cases of sarcoidosis, TNF-I have been reported effective for achieving resolution. However, the progression of sarcoidosis due to the TNF-I also has been reported. We herein report a case of pulmonary sarcoidosis with a Crohn’s disease (CD) patient developed after a long period administration (15 years) of TNF-I.

**Case presentations:**

A 37-year-old woman with CD who had been diagnosed at 22 years old had been treated with the TNF-I (original infliximab; O-IFX and infliximab biosimilar; IFX-BS). Fifteen years after starting the TNF-I, she developed a fever and right chest pain. Chest computed tomography (CT) revealed clustered small nodules in both lungs and multiple enlarged hilar lymph nodes. Infectious diseases including tuberculosis were negative. Bronchoscopic examination was performed and the biopsy specimens were obtained. A pathological examination demonstrated noncaseating granulomatous lesions and no malignant findings. TNF-I were discontinued because of the possibility of TNF-I-related sarcoidosis. After having discontinued for four months, her symptoms and the lesions had disappeared completely. Fortunately, despite the discontinuation of TNF-I, she has maintained remission.

**Conclusions:**

To our knowledge, this is the first case in which sarcoidosis developed after switching from O-IFX to IFX-BS. To clarify the characteristics of the cases with development of sarcoidosis during administration of TNF-I, we searched PubMed and identified 106 cases. When developing an unexplained fever, asthenia, uveitis and skin lesions in patients with TNF-I treatment, sarcoidosis should be suspected. Once the diagnosis of sarcoidosis due to TNF-I was made, the discontinuation of TNF-I and administration of steroid therapy should be executed promptly. When re-starting TNF-I, another TNF-I should be used for disease control. Clinicians should be aware of the possibility of sarcoidosis in patients under anti-TNF therapy.

**Supplementary Information:**

The online version contains supplementary material available at 10.1186/s12876-021-01948-6.

## Background

Inflammatory bowel disease (IBD) is chronic inflammation of the entire gastrointestinal tract, although its etiology has largely been unclear. Tumor necrosis factor (TNF) inhibitors are known to be effective treatment for treating IBD patients with moderate to severe activity. The cost-effectiveness and efficacy of TNF inhibitors (TNF-I) has been demonstrated through their reduction in the rates of hospitalization and surgery [1]. Recently, biosimilars of TNF-I, such as CT-P13, have been developed and are thought to possess equal efficacy and safety to the original with dramatic cost benefits. Switching from the original to a biosimilar is thus considered an acceptable treatment [2, 3].

Similar to IBD, sarcoidosis is a systemic granulomatous disease of unknown etiology, affecting various organs, including the lung, heart, lymphatic system and skin. In many cases of sarcoidosis, steroids are effective for treatment, and in case of steroid resistance, TNF-I are reported to be effective. While some studies have reported that the administration of TNF-I caused the progression of sarcoidosis, no reports regarding the relationship between sarcoidosis and infliximab biosimilar (IFX-BS) have been published.

We herein report a case of pulmonary sarcoidosis in a Crohn’s disease (CD) patient during fifteen years administration of IFX-BS after switching from original infliximab (O-IFX). To our knowledge, this is the first case of sarcoidosis developing after switching from O-IFX to IFX-BS in a CD patient.

## Case presentation

A 37-year-old Japanese woman was diagnosed with CD at 22 years of age. She had no relevant family history. At the onset of CD, she had symptoms of fever, abdominal pain, and frequent diarrhea. On total colonoscopy, she was found to have multiple longitudinal ulcers in the terminal ileum with stricture. Her symptoms were severe; thus, we administered O-IFX first, without steroid therapy. Clinical remission was obtained after 3 months of O-IFX treatment. She had maintained clinical remission without any adverse events for twelve years after the administration of O-IFX, and then O-IFX was switched to IFX-BS (CT-P13) after obtaining informed consent, because IFX-BS demonstrated equivalent efficacy and safety in the treatment of CD and the drug price was approximately half that of O-IFX in Japan. After switching to IFX-BS, clinical remission was still maintained for three years.

**Fig. 1:**
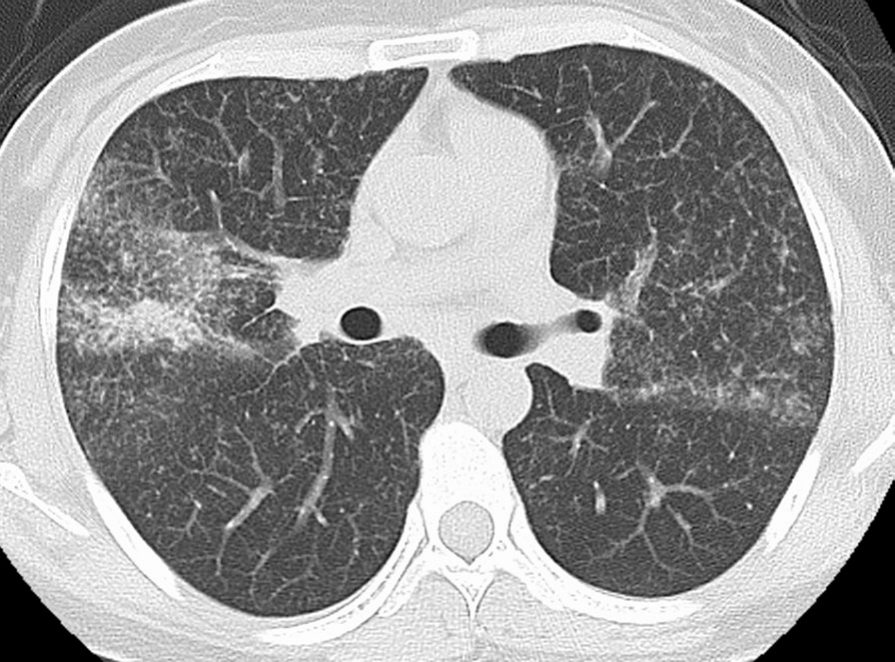
CT scan showing multiple nodular lung lesions and mediastinal and hilar lymphadenopathies

**Fig. 2:**
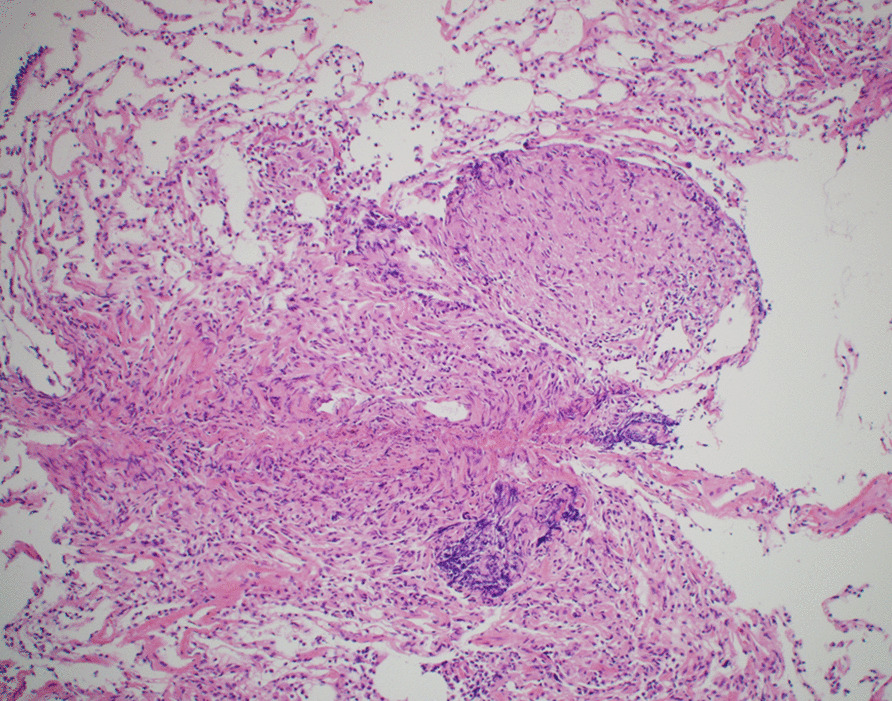
Histological findings of the lung. Histology demonstrated noncaseating granulomatous lesions and no malignant findings (X100)

**Fig. 3:**
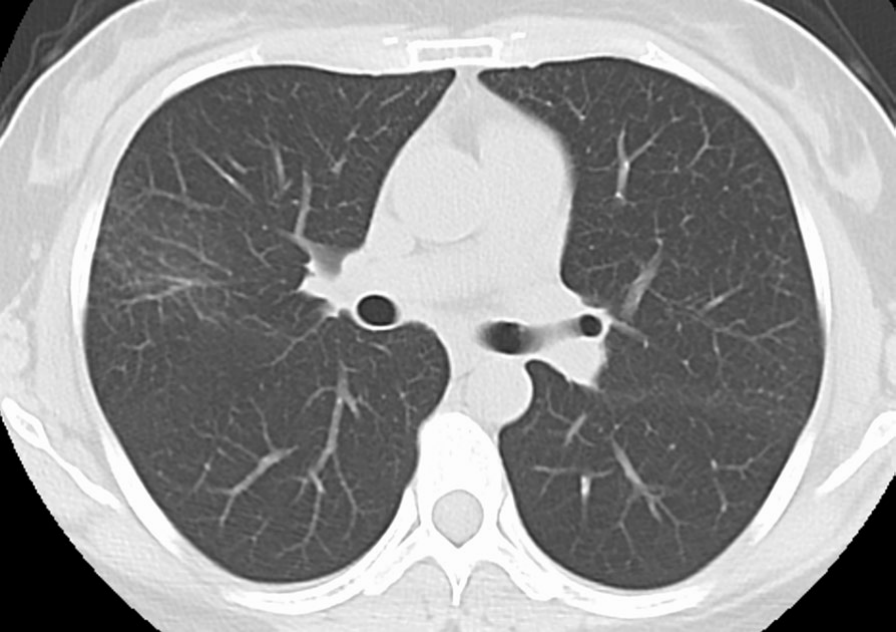
The nodular lung lesions disappeared after discontinuation of IFX-BS in CT scan

Fifteen years after starting the TNF-I (O-IFX and CT-P13), she developed a fever and right chest pain but had no respiratory symptoms, such as cough or sputum. Laboratory findings showed total bilirubin, 1.5 mg/dL; alanine aminotransferase, 248 U/L; aspartate aminotransferase, 105 U/L; gamma glutamyl aminotransferase, 192 U/L; alkaline phosphatase, 489 U/L; C-reactive protein (CRP), 0.44 mg/dL; anti-nuclear antibody, 1:160. Hepatitis B and C were negative. Chest X-ray and computed tomography (CT) revealed clustered small nodules in both lungs and multiple enlarged hilar lymph nodes (Fig. 1). The interferon gamma release assay and an acid-fast bacilli antibody were negative. A tuberculosis polymerase chain reaction (PCR) assay and bacterium culture of her sputum were both negative. Subsequently, a bronchoscopic examination was performed, and biopsy specimens were obtained from the middle and lower lobes of the right lung. A pathological examination demonstrated noncaseating granulomatous lesions and no malignant findings (Fig. 2). The serum angiotensin-converting enzyme and lysozyme levels were within normal limits, and soluble interleukin-2 receptor (sIL-2R) was increased to 627 U/mL (reference range 122–496 U/mL). Thus, the diagnosis of sarcoidosis was made. Her symptoms (low-grade fever and slight chest pain) could be sufficiently managed by the administration of an analgesic antipyretic. Therefore, we did not use steroids and, considering the possibility of TNF-I related sarcoidosis, TNF-I was discontinued. Subsequently, her symptoms improved gradually. In addition, the abnormal laboratory findings, including hepatobiliary enzymes, improved naturally without any treatment after one month. After having discontinued TNF-I for four months, her symptoms of sarcoidosis and the lesions detected by chest CT had disappeared completely (Fig. 3). Fortunately, despite the discontinuation of TNF-I, she has maintained clinical and endoscopic remission for 18 months after the discontinuation of TNF-I.

## Discussions and conclusions

We herein report the pulmonary sarcoidosis after the long-term use of TNF-inhibitors in a CD patient.

Two possible causes were considered responsible for the development of sarcoidosis in our case: the long-term IFX administration and the switching from the original agent to its biosimilar. To confirm the involvement of these causes and clarify the characteristics of patients likely to develop sarcoidosis under TNF-I therapy, we searched for case reports in PubMed concerning sarcoidosis due to TNF-I using the combination of the heading terms ‘infliximab’, ‘adalimumab’, ‘etanercept’, ‘certolizumab’, ‘golimumab’, ‘infliximab biosimilar’, with or without ‘Crohn’s disease’, combined with ‘sarcoidosis’. We identified 6 articles concerning 7 cases using the word combination with CD [4–9] and 65 articles concerning 99 cases using the word combination without CD (details in Additional file 1: Table 1) [10–74]. Regarding the impact of long-term TNF-I administration, in 103 cases, the median duration of developing sarcoidosis was 21 (range 1–90) months, and the maximum period was 90 months, according to previous reports. However, since those reports contained various diseases, we reviewed the seven cases with CD (excluding our own case). The median duration to the development of sarcoidosis was 24 (range 7–90) months, and the maximum period was 90 months. Our case was administered O-IFX for 144 months and then IFX-BS for 36 months. Based on the previous reports, the duration of 180 months until the development of sarcoidosis in our case was the longest, exceeding the longest period in previous reports by more than 7 years. Long-term IFX administration may affect the development of sarcoidosis, as the administration of TNF-I presumably induces cytokine imbalance (TNF-α suppression and excessive Interferon-α production) [75]. It took a very long time to for the patient to develop sarcoidosis; however, it may have been a possible complication as the time to the induction of cytokine imbalance seems to show individual differences. In addition, infection is suggested to be a cause of sarcoidosis, and the treatment with TNF-I increases the risk of various infections [10–12], which can lead to the onset of sarcoidosis. Prudent follow-up should thus be performed, including observation of CD patients under long-term IFX administration. Regarding the impact of switching from O-IFX to IFX-BS, we thought that the possibility of sarcoidosis caused by changing O-IFX to IFX-BS would be quite low because of the very slight differences in the sugar chain sequences of O-IFX and IFX-BS and their almost equivalent immunogenicity [76].

Regarding other characteristics of CD patients, the median age was 30 (range 21–44) years, and the male:female ratio was 5:3 when including our case. The causative TNF inhibitors were adalimumab (ADA) (n = 4, 50.0%, including a case switching from IFX to ADA) and IFX (n = 4, 50%, including our case). The symptoms depended on the organs involved. Our case showed an unexplained fever and chest pain because of pleuritis. Asthenia, uveitis, and skin lesions were also reported. There were no specific symptoms.

In this case, the serum ACE level was normal. It was reported that serum ACE shows low sensitivity and specificity in the diagnosis of sarcoidosis [77]. In addition, it is known that the serum ACE level is affected by genetic polymorphism [78, 79]. Clinicians should recognize that the serum ACE levels alone are not enough to diagnose sarcoidosis.

We also investigated the difference in the duration until the development of sarcoidosis under IFX and ADA therapy in the six CD cases without switching cases. The median duration to the development of sarcoidosis in CD patients under IFX (n = 3) and ADA (n = 3) therapy was 12 and 24 months, respectively. Patients receiving ADA therapy showed a longer duration until development than those receiving IFX, which might have influenced the difference in antibody type, such as chimeric human-mouse IgG monoclonal antibody and fully human monoclonal antibody. However, the number of such cases has been limited so far, so the accumulation of more cases will be required to verify whether or not differences in antibodies affects the development of sarcoidosis.

Regarding the therapy delivered after the development of sarcoidosis, six of eight cases discontinued TNF-I, two without steroid and four with additional steroid, and two cases continued TNF-I therapy while adding steroid therapy or topical therapy. All six cases who discontinued TNF-I with/without steroids showed the resolution of sarcoidosis. In one case, in which the patient continued TNF-I with steroid treatment, improvement of the lung lesion was observed. In one case in which the patient continued TNF-I with topical steroid treatment, improvement of the lung lesion, but not the skin lesion, was observed. In CD patients who develop sarcoidosis, discontinuation of TNF-I seems to be a feasible treatment. Among 107 cases with various diseases, 47 discontinued TNF-I without the addition of steroid therapy, and sarcoidosis was improved or resolved in 43 (91.5%), while 49 cases discontinued TNF-I with the addition of steroid therapy, and 46 (93.9%) showed improvement or resolution of sarcoidosis. These data support the notion that discontinuing TNF-I is effective for treating patients with chronic inflammatory disease, but the efficacy of additional steroid therapy is unclear.

Our case has maintained remission despite having discontinued TNF-I; however, TNF-I may be key drugs for CD patients. Thus, whether or not TNF-I can be re-initiated for these patients is important to clarify. Only one CD patient re-initiated the same TNF inhibitor (O-IFX) with no recurrence of sarcoidosis observed [8]. When expanding target diseases to include rheumatoid arthritis, ankylosing spondylitis, psoriatic arthritis, spondyloarthropathy, psoriasis, CD, juvenile idiopathic arthritis, SAPHO (synovitis, pustulosis, acne, hyperostosis, osteitis), ulcerative colitis, 25 patients re-initiated TNF-I. Of 25 patients, 7 were administered the same TNF-I, and sarcoidosis consequently recurred in 4 cases (57.1%) [8, 11, 23, 27, 39, 63]. Eighteen cases started another TNF-I, and sarcoidosis recurred in 3 cases (16.7%) [13, 27, 64–74]. These data suggested that another TNF-I should be administered when re-initiating TNF-I in such patients.

In conclusion, should be aware that TNF-I can cause several drug related complications, including sarcoidosis. Sarcoidosis due to TNF-I can be caused by IFX-BS and develop even after long-term administration. Clinicians should be aware of the possibility of sarcoidosis in patients under anti-TNF therapy. The efficacy of additional steroid therapy remains unclear; however, TNF-I should be discontinued as soon as possible, even in CD patients who maintain long-term remission with TNF-I. When re-starting TNF-I, another TNF-I should be used for disease control, as relapse of sarcoidosis is frequent when patients are treated with the same TNF-I (Table 1).

**Table 1:** The characteristics of sarcoidosis in CD patients receiving TNF inhibitor therapy

References	Sex	TNF inhibitor/delay until onset of causative TNF inhibitor (months)	Organs involved	Treatment/outcome	Re-initiation TNF inhibitor	Relapse after re-initiation
[4]	F	IFX/60	Spinal cord	IFX discontinuation and PSL/resolution	–	
[5]	M	ADA/24	Hilar and mediastinal nodes, erythema nodosum	ADA discontinuation and PSL/resolution	–	
[6]	F	ADA/18	Mediastinal nodes, nodules on the spleen	ADA continue and PSL/resolution	–	
[7]	M	IFX/7	Erythematous skin plaques, bilateral nodular pulmonary infiltrates	IFX continue and topical therapy /resolution of lung, but skin not resolved	–	
[8]	M	IFX/12	hilar adenopathies, pulmonary infiltrate	IFX discontinuation/resolution	IFX re-start	
[9]	M	ADA/60	Uveitis, mediastinal nodes, mesenterial nodes	ADA discontinuation and PSL/resolution	–	
	M	IFX/72, ADA/18	Erythema nodosum, mediastinal nodes	ADA discontinuation and PSL/resolution	–	
Present case	F	O-IFX 144, IFX-BS 36	Bilateral pulmonary infiltrates, hilar and mediastinal lymphadenopathy	IFX-BS discontinuation/resolution	–	

M, male; F, female; ETN, etanercept; ADA, adalimumab; IFN, infliximab; IFX-BS, infliximab biosimilar

## Supplementary Information

**Additional file 1**. To confirm the involvement of these causes and clarify the characteristics of patients likely to develop sarcoidosis under TNF inhibitor therapy, we searched for case reports in PubMed concerning sarcoidosis due to TNF inhibitor using the combination of the heading terms ‘infliximab’, ‘adalimumab’, ‘etanercept’, ‘certolizumab’, ‘golimumab’, ‘infliximab biosimilar’, without ‘Crohn’s disease’, combined with ‘sarcoidosis’.

## Data Availability

The datasets used and/or analyzed during the current study available from the corresponding author on reasonable request.

## Abbreviations

IBD: Inflammatory bowel disease
TNF: Tumor necrosis factor
TNF-I: TNF inhibitor
CD: Crohn’s disease
O-IFX: Original infliximab
IFX-BS: Infliximab biosimilar
CT: Computed tomography
ADA: Adalimumab
