# Supplementary material for: Development of pulmonary sarcoidosis in Crohn’s disease patient under infliximab biosimilar treatment after long-term original infliximab treatment: a case report and literature review
Source: BMC Gastroenterol. 2021 Oct 12;21:373. doi: 10.1186/s12876-021-01948-6 (PMC8513323; doi:10.1186/s12876-021-01948-6)
Supplement: Supplementary file 1 — Additional file 1. To confirm the involvement of these causes and clarify the characteristics of patients likely to develop sarcoidosis under TNF inhibitor therapy, we searched for case reports in PubMed concerning sarcoidosis due to TNF inhibitor using the combination of the heading terms ‘infliximab’, ‘adalimumab’, ‘etanercept’, ‘certolizumab’, ‘golimumab’, ‘infliximab biosimilar’, without ‘Crohn’s disease’, combined with ‘sarcoidosis’. [file 12876_2021_1948_MOESM1_ESM.docx]

| reference | disease | sex | TNF inhibitor/ Delay until onset of causative  TNF inhibitor (months) | Organs involved | treatment/outcome | Re-initiation  TNF inhibitor | Relapse  after  re-initiation |
| --- | --- | --- | --- | --- | --- | --- | --- |
| 10 | JIA | F | ADA/1 | cutaneous nodules | ADA discontinuation and PSL/resolution | - |  |
|  | PsA | M | IFX/4 | cutaneous nodules | IFX discontinuation/resolution | - |  |
| 11 | RA | F | ADA/8 | Erythema nodosum,  mediastinal nodes | ADA discontinuation/resolution | ADA re-start | - |
|  | RA | F | ETN/60 | Parotic gland, cervical lymphadenopathy,  hilar nodes | ETN discontinuation and PSL/resolution | ETN re-start | - |
| 12 | RA | F | ETN/26 | hilar adenopathies and pulmonary infiltrate | ETN discontinuation/resolution | - |  |
|  | AS | M | IFX/22 | intrathoracic adnopathies | IFX discontinuation/resolution | - |  |
| 13 | SAPHO | F | ADA/1 | pulmonary infiltrate, mediastinal and hilar nodes,  nodular long lesions | ADA discontinuation/resolution | - |  |
|  | RA | F | ADA/21 | Skin, pulmonary infiltrate, mediastinal nodes | ADA discontinuation/resolution | - |  |
|  | AS | M | IFX/51 | Mediastinal and hilar nodes | IFX discontinuation /Clinical but not radiological resolution | - |  |
|  | RA | F | ETN/27 | Skin, uvea, apex lobes infiltrate, hilar nodes | ETN discontinuation and PSL/resolution | ADA start | - |
|  | PsA | M | ETN/2 | Skin | ETN discontinuation/resolution | - |  |
|  | RA | F | ETN/18 | Skin | ETN discontinuation/resolution | - |  |
|  | RA | F | ETN/26 | bilateral pulmonary reticulonodular infiltrates,  hilar nodes | ETN discontinuation/resolution | - |  |
|  | AS | F | ETN/18 | bilateral pulmonary infiltration | ETN discontinuation/resolution | ADA start | + |
|  | AS | M | IFX/17 | Bilateral pulmonary nodular infiltrates,  mediastinal nodes | IFX discontinuation/resolution | - |  |
|  | AS | F | IFX/14 | Skin, pulmonary infiltrates,  mediastinal nodes | IFX discontinuation/resolution | ETN start | - |
| 14 | PsA | M | IFX/22 | Bilateral pulmonary reticulonodular infiltrates,  hilar nodes | IFX discontinuation and PSL/resolution | - |  |
|  | RA | F | ADA/27 | Bilateral pulmonary infiltrates | ADA discontinuation and PSL/resolution | - |  |
| 15 | SpA | F | ADA/8 | Erythema nodosum, mediastinal nodes | ADA discontinuation/resolution | - |  |
|  | RA | F | ADA/48 | Mediastinal nodes | ADA discontinuation and PSL/resolution | - |  |
| 16 | RA | F | ETN/9 | Hilar nodes, cardiac failure | ETN discontinuation and PSL/resolution | - |  |
| 17 | RA | F | ETN/60 | Acute renal failure,  bilateral interstitial lung infiltration,  splenomegaly | ETN discontinuation and PSL/resolution | - |  |
| 18 | RA | F | ETN/84 | Lymphocytic meningitis,  bilateral peripheral facial paralysis and anosmia,  hilar lymph, uveitis | ETN discontinuation and PSL/resolution | - |  |
| 19 | JIA | M | ETN/3 | Skin rash, vitritis | ETN discontinuation and PSL/resolution | - |  |
| 20 | PsA | M | ADA/72 | Hypofaryngeal infiltration, tougne ulcer, cervical lymphadenopathy | ADA discontinuation and PSL/resolution | - |  |
| 21 | RA | F | ETN/6 | Infiltrated erythematous skin lesions,  hilar nodes, augmentation of parotids | ETN discontinuation and PSL/resolution | - |  |
| 22 | SpA | F | ETN/12 | Cutaneous granulomatous lesions | ETN discontinuation/resolution | - |  |
| 23 | RA | F | ADA/3 | Mediastinal and hilar nodes, skin, joints | ADA discontinuation and PSL/resolution | - |  |
|  | RA | M | ETN/8, ADA/3 | Mediastinal and hilar nodes | ADA discontinuation and PSL/not resolved | - |  |
|  | PsA | F | ETN/36 | Interstitial nefritis | ETN discontinuation and PSL/resolution | ETN re-start | + |
| 24 | UC | F | IFX/23 | Prominence of previous scars and dermal nodules | IFX continue and PSL/resolution | - |  |
| 25 | RA | F | ADA/47 | Panuveitis, papular skin lesion | ADA discontinuation and PSL/not resolved | - |  |
| 26 | PsA | F | ETN/48 | Anterior uveitis,  mediastinal and hilar nodes | ETN discontinuation and PSL/resolution | - |  |
| 27 | RA | F | ETN/36 | Hilar nodes | ETN discontinuation and PSL/resolution | - |  |
|  | JIA | F | ETN/24 | Lacrimal and salivary glands,  dryness of month and eyes | ETN discontinuation/resolution | ETN re-start | + |
|  | AS | M | ETN/12 | Uveitis, cervical lymph nodes,  lacrimal en salivary glands,  liver granulomas | ETN discontinuation and PSL/resolution | ADA start | - |
| 28 | RA | M | ADA/36, ETN/6 | Erythema nodosum, ankle synovitis,  hilar lymph nodes,  interstitial nodular changes | ETN discontinuation/resolution | - |  |
| 29 | RA | F | ETN/49 | reticular-nodular pulmonary infiltrates | ETN discontinuation and PSL/resolution | - |  |
| 30 | RA | F | ADA/11 | Parotic gland, cervical lymph nodes | ADA discontinuation/resolution | - |  |
| 31 | AS | M | ETN/24 | Right upper lobe infiltrate, mediastinal nodes | ETN discontinuation and PSL/resolution | - |  |
| 32 | RA | F | ADA/21 | Cervical, axillary and inguinal nodes,  bone marrow | ADA discontinuation and PSL/resolution | - |  |
| 33 | PsA | F | ETN/7 | Hepatic granulomas | ETN discontinuation/resolution | - |  |
| 34 | RA | F | ETN/36 | Pulmonary nodules, diffuse lymphadenopathy | ETN discontinuation and PSL/resolution | - |  |
|  | RA | F | IFX/60 | Anterior uveitis, erythema nodosum,  cutaneous rash, hilar and mediastinal nodes,  fibrotic lung disease | IFX discontinuation and PSL/resolution | - |  |
|  | RA | F | ETN/24 | Erythema nodosum, interstitial lung disease,  hilar and mediastinal nodes | ETN discontinuation/resolution | - |  |
| 35 | PS | M | ETN/9 | Hilar and mediastinal nodes | ETN discontinuation/resolution | - |  |
| 36 | PS | F | ADA/5 | Subcutaneous nodules,  hilar lympadenopathy | ADA discontinuation/resolution | - |  |
| 37 | RA | F | ETN/14 | Hilar and mediastinal nodes,  abnormal uptake by gallium scan in the salivary glands, paratracheal region and right quadriceps muscle,  granulomata in skin and liver | ETN discontinuation and PSL/resolution | - |  |
| 38 | PsA | M | ETN/2 | Erythematous non-tender skin nodules,  mediastinal and hilar nodes | ETN discontinuation/resolution | - |  |
| 39 | RA | M | ADA/12 | Asthenia, cavitated pulmonary lesion | ADA discontinuation/resolution | - |  |
|  | RA | M | ADA/17 | Pulmonary nodules | ADA discontinuation/resolution | ADA re-start | + |
|  | RA | M | ETN/6 | Pulmonary nodules | ETN discontinuation and Rituximab/resolution | - |  |
|  | RA | F | ETN/9 | Bilateral granulomatous pulmonary nodules | ETN discontinuation and Rituximab/resolution | - |  |
|  | RA | F | ADA/12 | Pulmonary nodules | ADA continue/no progression of the nodules | - |  |
|  | RA | M | ETN/13 | hilar adenopathies | ETN discontinuation /no progression of the nodules | - |  |
|  | RA | F | ETN/24 | Bilateral pulmonary nodules | ETN continue/no progression of nodules | - |  |
|  | RA | M | ETN/31 | nodular pulmonary granulomas | ETN discontinuation /no progression of the nodules | - |  |
|  | RA | M | ETN/36 | Apical pulmonary nodules | ETN continue/no progression of nodules | - |  |
|  | RA | M | IFX/48 | Bilateral pulmonary nodules | IFX continue/no progression of the nodules | - |  |
|  | RA | M | IFX/48 | Bilateral pulmonary nodules | IFX discontinuation /no progression of the nodules | - |  |
| 40 | RA | F | ETN/not mention | Eyes (uveitis) and hilar lymph nodes | ETN discontinuation and PSL/resolution | - |  |
| 41 | RA | F | IFX/33 | Skin, laterotracheal nodes and micro nodular lesions | IFX discontinuation/resolution | - |  |
| 42 | RA | F | ETN/20 | Lungs, hilar and mediastinal nodes | ETN discontinuation/resolution | - |  |
| 43 | AS | F | ETN/1 | Lungs, hilar and mediastinal nodes, uvea | ETN discontinuation and PSL/resolution | - |  |
| 44 | RA | F | ETN/19 | Hepatic granulomas | ETN discontinuation/resolution | - |  |
| 45 | SpA | F | ETN/10 | Lungs | ETN discontinuation and PSL/resolution | - |  |
| 46 | RA | F | IFX/68 | Central nervous system | IFX discontinuation and PSL/resolution | - |  |
| 47 | RA | F | ETN/12 | Skin, mediastinal nodes | ETN discontinuation/resolution | - |  |
|  | RA | F | ETN/6 | Lungs, hilar and mediastinal nodes | ETN discontinuation and PSL/resolution | - |  |
| 48 | AS | M | IFX/28 | hilar and mediastinal lymphadenopathy | IFX discontinuation/resolution | - |  |
| 49 | AS | M | ETN/1 | Erythematous and papular skin lesions in a tattoo | ETN continue and PSL/resolution | - |  |
| 50 | RA | F | ETN/18 | Parotic gland swelling,  mediastinal and bronchopulmonary nodules | ETN discontinuation and PSL/resolution | - |  |
| 51 | AS | M | ETN/21 | Skin, hilar and paratracheal nodes,  parotid and lacrimal glands | ETN discontinuation/resolution | - |  |
| 52 | AS | M | IFX/60 | Pleural effusion, mediastinal and hilar nodes | IFX discontinuation and PSL /Clinical but not radiological resolution | - |  |
| 53 | JIA | M | ETN/1 | cutaneous nodule, uveitis | ETN discontinuation and PSL/resolution | - |  |
| 54 | RA | F | ETN/22 | Subpleural nodule and a cavitated lesion | ETN discontinuation/resolution | - |  |
| 55 | RA | F | ETN/2 | Skin lesions, diffuse reticulonodular pulmonary infiltrate | ETN discontinuation and PSL/resolution | - |  |
| 56 | UC | M | ADA/12 | pulmonary nodules and renal dysfunction | ADA discontinuation and PSL/resolution | - |  |
| 57 | PS | M | ADA/12 | pulmonary nodules,  bilateral hilar and mediastinal adenopathy | ADA discontinuation /no progression of the nodules | - |  |
| 58 | PsA | M | ADA/24 | brain, hilar and mediastinal lymphadenopathy,  small pulmonary nodules, liver ocoid lesion | ADA discontinuation/resolution | - |  |
| 59 | PS | M | ETN/24, ADA/12 | intestinal lymphadenopathies | ADA discontinuation/resolution | - |  |
| 60 | AS | M | ADA/10 | pulmonary nodules,  hilar and mediastinal lymphadenopathies | ADA discontinuation/resolution | - |  |
| 61 | UC | M | IFX/44 | pulmonary micronodules,  mediastinal lymphadenopathies, retroperitoneal lymphadenopathies | IFX discontinuation and PSL/resolution | - |  |
| 62 | PsA | F | ADA/not mention | orbital edema | ADA discontinuation and PSL/resolution | - |  |
| 63 | PsA | M | ADA/12 | cutaneous nodule, bilatetal hilar lymphadenopathy,  pulmonary patchy consolidation | ADA discontinuation and PSL/resolution | ADA re-start | + |
| 64 | RA | F | ETN/not mention | Uveitis | ETN discontinuation/resolution | ADA start | - |
| 65 | AS | M | ADA/60 | Seizures | ADA discontinuation and PSL/resolution | ETN start | - |
| 66 | RA | F | ETN/48 | Subcutaneous nodules,  pretracheal, subcarinal, hilar and peribronchial nodes | ETN discontinuation/resolution | ADA start | - |
| 67 | PsA | M | IFX/48 | Mediastinal and hilar nodes,  interstitial pulmonary changes, interstitial nephritis | IFX discontinuation and PSL/resolution | ETN start | + |
| 68 | RA | F | ETN/60 | Ground-glass pulmonary opacities,  mediastinal nodes | ETN discontinuation/resolution | ADA start | - |
|  | RA | M | ETN/9 | Pulmonary nodules | ADA discontinuation and PSL/resolution | ADA start | - |
|  | RA | F | ETN/48 | Pleural effusions, interstitial lung disease and nodules | ETN discontinuation and PSL/resolution | ADA start | - |
|  | RA | F | ETN/48 | Diffuse pulmonary micronodules, mediastinal nodes | ETN discontinuation and PSL/resolution | ADA start | - |
| 69 | SpA | F | IFX/7 | Erythemato-squamous and nodular skin lesions,  deep vein thrombosis, pulmonary ground-glass,  subcarinal nodes | IFX discontinuation/resolution | ETN start | - |
| 70 | PsA | M | ETN/24 | bilateral interstitial reticulonodular pulmonary infiltrates | ETN discontinuation/resolution | ADA start | - |
| 71 | AS | F | IFX/18 | skin, bilateral hilar and mediastinal lymphadenopathy | IFX discontinuation/resolution | ADA start | - |
| 72 | JIA | F | ETN/24 | brain and mediastinal lymphadenopathy | ETN discontinuation and PSL/resolution | IFX start | - |
| 73 | AS | M | ADA/10 | Pulmonary nodules, hilar and mediastinal lymphadenopathy | ADA discontinuation/resolution | ETN start | - |
| 74 | RA | F | ETN/2 | Erythema nodosum, hilar nodes | ETN discontinuation and PSL/resolution | IFX start | + |

**Supplementary table:**

**M = male, F = female, RA = rheumatoid arthritis, AS = ankylosing spondylitis, PsA = psoriatic arthritis, SpA = spondyloarthropathy, PS = psoriasis, CD= Crohn’s disease,**

**JIA = juvenile idiopathic arthritis, SAPHO = synovitis, pustulosis, acne, hyperostosis, osteitis, UC = ulcerative colitis, ETN = etanercept, ADA = adalimumab, IFN = infliximab,**

**IFX-BS = infliximab biosimilar.**
